# A dual role of Cdk2 in DNA damage response

**DOI:** 10.1186/1747-1028-4-9

**Published:** 2009-05-18

**Authors:** Ande Satyanarayana, Philipp Kaldis

**Affiliations:** 1 Institute of Molecular and Cell Biology (IMCB), Proteos, 61 Biopolis Drive, Singapore 138673, Republic of Singapore; 2 Mouse Cancer Genetics Program, Center for Cancer Research, National Cancer Institute-Frederick, Bldg 560/22-69, 1050 Boyles Street, Frederick, Maryland 21702-1201, USA

## Abstract

Once it was believed that Cdk2 was the master regulator of S phase entry. Gene knockout mouse studies of cell cycle regulators revealed that Cdk2 is dispensable for S phase initiation and progression whereby Cdk1 can compensate for the loss of Cdk2. Nevertheless, recent evidence indicates that Cdk2 is involved in cell cycle independent functions such as DNA damage repair. Whether these properties are unique to Cdk2 or also being compensated by other Cdks in the absence of Cdk2 is under extensive investigation. Here we review the emerging new role of Cdk2 in DNA damage repair and also discuss how the loss of Cdk2 impacts the G_1_/S phase DNA damage checkpoint.

## Introduction

Cyclin dependent kinases (Cdks), a family of serine/threonine kinases, faithfully control the mammalian cell cycle by binding to cyclins [1]. Under normal circumstances, D-type cyclins activate Cdk4 and/or Cdk6 and initiate phosphorylation of Retinoblastoma protein (Rb) family early in the G_1 _phase [2,3]. This leads to the release of E2F transcription factors and results in activation of transcription of E2F responsive genes required for cell cycle progression [4,5]. In the late G_1 _phase, cyclin E activates Cdk2 [2,3] and completes the phosphorylation of Rb leading to passage through the restriction point at the boundary of the G_1_/S phase, and to S phase initiation. Later Cdk2 plays an important role in S phase progression by complexing with cyclin A. Finally Cdk1/cyclin B complexes actively participate and complete mitosis [6]. Although it was believed that Cdk2 was essential for S phase entry [7], recent studies found that Cdk2 is dispensable for S phase initiation and progression in vivo and Cdk1 can take over Cdk2's function in its absence [8-10]. Moreover, it was discovered that Cdk1 alone can drive progression of the mammalian cell cycle by complexing with all the phase specific cyclins in the absence of Cdk2, Cdk4, and Cdk6 [11].

Cdk/cyclin complexes, due to their major role in cell cycle regulation, have become prime targets of inhibition during cellular stress, DNA damage, and telomere dysfunction [12,13]. Two families of Cdk inhibitors regulate the activities and functions of Cdk/cyclin complexes. The INK4 family (p16, p15, p18, p19) specifically bind to Cdk4/Cdk6 and prevent D-type cyclin activity, whereas the Cip/Kip family (p21, p27, p57) inhibits Cdk2/cyclin E, Cdk2/cyclin A as well as Cdk1/cyclin B activity [2,12,14,15]. In response to DNA damage, cell cycle checkpoints (G_1_, S, G_2_/M) are activated and stop cell cycle progression to allow time for repair thereby preventing transmission of damaged or incompletely replicated chromosomes [12,16]. Cdk2/cyclin E complexes are inhibited to arrest cells at the G_1_/S checkpoint whereas Cdk1/cyclin B complexes are inhibited to arrest the cells at G_2_/M phase of the cell cycle [12].

## Cdk2 in the DNA damage checkpoint and DNA repair

**Figure 1:**
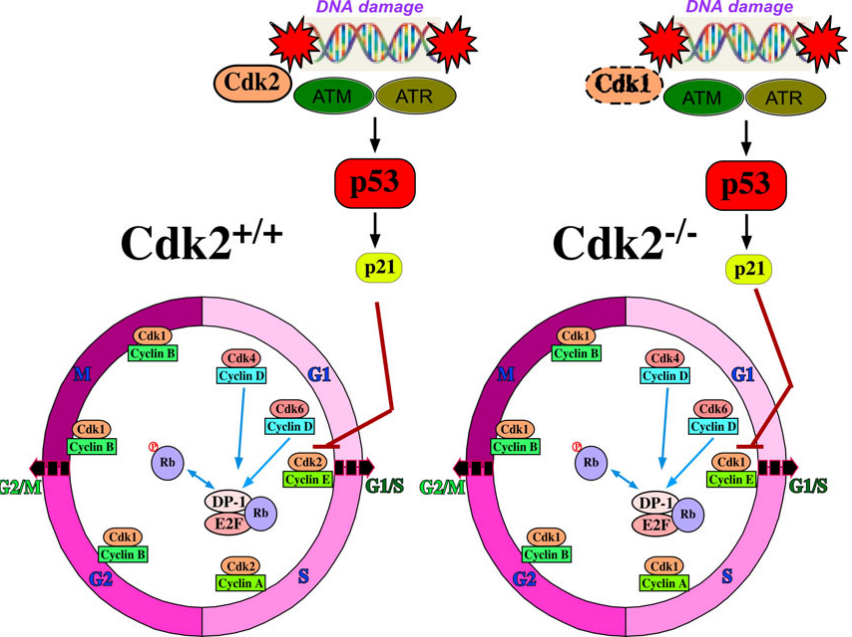
**Maintenance of the G_1_/S DNA damage checkpoint in the presence and absence of Cdk2**. In response to DNA damage, activation of p53-p21 pathway is not altered in the absence of Cdk2, the primary target of p21 at the G_1_/S checkpoint. In the presence of Cdk2, the induced p21 inhibits Cdk2/cyclin E complexes in response to DNA damage. In the absence of Cdk2, Cdk1/cyclin E complexes are responsible for promotion of the G_1_/S transition and as a result become the target for p21 inhibition in response to DNA damage. Nevertheless, Cdk1 is not fully capable to rescue the functions of Cdk2 in DNA damage repair.

The G_1 _cell cycle checkpoint is primarily responsible for preventing damaged DNA from being replicated. During G_1_/S DNA damage checkpoint arrest, ATM/ATR mediated activation of p53 activates one of its downstream targets, p21^Cip1/Waf1 ^[17]. p21 binds to and inhibits Cdk2/cyclin E complexes thereby arresting cells at the G_1_/S transition. As a result, Cdk2 has become the prime target of the G_1_/S DNA damage checkpoint and Cdk2 inhibition by p21 is one essential step in maintaining the G_1_/S DNA damage checkpoint [12,17] (Figure 1). Nevertheless, the loss of Cdk2 presents a different challenge to cells, which in turn might lead to altered DNA damage response and checkpoint activation. An important issue that was ignored with regard to Cdk2's substitution by Cdk1 is how the G_1_/S DNA damage checkpoint functions in the absence of Cdk2. In this regard the first question that arises, is how the p53 pathway operates and how is p21 regulated in the absence of its target Cdk2? If p21 is induced and the cells arrest at the G_1_/S checkpoint, which target will be inhibited by p21 in the absence of Cdk2? In this context, the finding that Cdk1/cyclin E substitutes for Cdk2/cyclin E presents an interesting twist to the G_1_/S DNA damage checkpoint especially due to the fact that Cdk1 is the prime target for p21 at the G_2_/M DNA damage checkpoint [12,18]. In the absence of Cdk2, will Cdk1 be able to regulate both G_1_/S and G_2_/M DNA damage checkpoints? Or do the cells bypass the G_1_/S checkpoint in the absence of Cdk2 and arrest only at the G_2_/M checkpoint? If Cdk1 is able to maintain the G_1_/S checkpoint, will Cdk1 be inhibited by p21 or is it phosphorylated at the inhibitory sites Thr14/Tyr15 and restricted to the cytoplasm that occurs under certain circumstances during G_2_/M DNA damage checkpoint arrest? As a result it was of interest to reanalyze the translocation pattern of Cdk1 in response to DNA damage especially at the G_1_/S transition in the absence of Cdk2. Another important question is, if p21 would be able to arrest cells at the G_1_/S transition even in the absence of Cdk2, will this arrest be persistent enough for the cells to repair their damaged DNA and resume DNA replication?

In a recent study it was shown that the activation of p53-p21 pathway is not perturbed in the absence of Cdk2 after DNA damage. The timing of induction of both p53 and p21 is similar in the presence or absence of Cdk2. In addition, cells were arrested promptly at the G_1_/S checkpoint in the absence of Cdk2 in response to irradiation induced DNA damage [19]. This indicates that p21 is able to arrest cells even in the absence of Cdk2. This observation suggests that p21 can block cell cycle progression by interacting with proteins other than Cdk2. In accordance with this hypothesis, it was revealed that the translocation of Cdk1 to the nucleus was not affected by irradiation induced DNA damage. As a result of premature translocation of Cdk1 to the nucleus, it co-localized with the induced p21 and was consequently inhibited by p21 at the G_1_/S DNA damage checkpoint. This indicates that in the absence of Cdk2, Cdk1 is responsible for driving the cells through the G_1_/S transition, which in turn is inhibited by p21 in response to DNA damage to maintain the G_1_/S checkpoint [19].

It was observed that even in the absence of Cdk2, cells were arrested for prolonged periods of time similar to wild type cells in response to irradiation. Moreover, in *Cdk2*^-/- ^cells the resumption of DNA replication was delayed after DNA damage in comparison to *Cdk2*^+/+ ^cells [19]. Similarly the *Cdk2*^-/-^ mice displayed increased sensitivity in response to irradiation and died earlier than wild type mice. Two possible mechanisms could explain this phenomenon, one is, perhaps loss of Cdk2 renders cells more sensitive to irradiation and as a result the cells have accumulated more extensive DNA damage in comparison to wild type cells. Another possibility is that Cdk2 might possess cell cycle independent functions in DNA repair and as a result *Cdk2*^-/- ^cells display delayed resumption of DNA replication due to impaired DNA repair activity. In accordance with this hypothesis, more extensive DNA damage was found in *Cdk2*^-/- ^cells. In addition, a delayed and impaired DNA repair activity was detected in *Cdk2*^-/- ^cells but it appears that *Cdk2*^-/- ^cells do not completely fail to form DNA repair foci (γH2AX, ATM/ATR substrates) at the sites of damage but rather they exhibited lower numbers of foci for prolonged periods of time [19].

In this context it would be interesting to explore the specific role of Cdk2 in DNA repair. Is Cdk2 part of the DNA repair complex or does Cdk2 play a more indirect role by initiating a signal transduction cascade or activating proteins that are involved in DNA repair? In support of this hypothesis, it was reported that purified Cdk2/cyclin A can phosphorylate S3291 of BRCA2 in vitro [20] and when Cdk2 activity is inhibited by Roscovitine, S3291 phosphorylation of BRCA2 is inhibited. This function is similar to that of Cdk1/cyclin B complexes that are responsible for phosphorylating S3291 of BRCA2 in mitosis, which in turn is necessary for RAD51 binding and DNA repair [21]. Moreover it was shown that cyclin A1 functions in DSB repair and this repair function is dependent on Cdk2 activity [22]. It appears that cyclin A1 is essential for the DNA repair function which in turn depends on the availability of Cdk2. As a result cyclin A1 fails to participate in DNA repair in the absence of Cdk2. This could be due to the lack of a direct interaction between cyclin A1 and Cdk1 as evident from the testis of Cdk2 knockout mice (no compensation of sterility [9,10]). It has been reported that inhibition of Cdk2 by Roscovitine leads to blunted activation of Chk1 due to reduced phosphorylation [22,23]. Chk1 is one of the downstream targets of the ATM/ATR pathway whose phosphorylation and activation is one of the necessary steps in the DNA damage signal transduction cascade since activated Chk1 in turn activates and promotes several DNA repair proteins to the sites of damage [24]. Two possible mechanisms could explain this phenomenon: 1) Cdk2 might phosphorylate Chk1 along with ATM/ATR there by accelerating the phosphorylation and activation of Chk1. In the absence of Cdk2 the activation process of Chk1 could be delayed resulting in delayed DNA repair; 2) Alternatively Cdk2 phosphorylates Chk1 at sites other than the sites which are phosphorylated by ATM/ATR and phosphorylation of these sites could be essential to complete activation of Chk1.

From the above studies it appears that Cdk2 acts upstream in the DNA damage pathway and is involved in the activation of DNA repair proteins (Figure 1). This is quite different from the downstream role of inhibited Cdk2 in arresting the cell cycle at the checkpoint. An interesting question to answer in this context is how does Cdk2 promote the activation of DNA repair proteins when it is present in the inhibited state and its kinase activity is inhibited by p21? Maybe, Cdk2 can promote DNA repair activity by direct interactions with other proteins that are involved in double strand break repair. In the inhibited state, Cdk2 is already in complex with cyclin E and/or cyclin A and p21, leaving little interaction space on the surface of the Cdk2 molecule. The observation that Cdk2 deficient cells display impaired DNA repair capacity indicates that Cdk1 might be unable to perform the DNA repair functions of Cdk2 in the absence of Cdk2 although it is able to trigger the G_1_/S DNA damage checkpoint. Another possibility could be that in response to DNA damage in the presence of Cdk2, p21 mainly targets Cdk2 (and only a few Cdk1 molecules) leaving a sub-pool of active Cdk1 that plays a role in DNA repair. This sub-pool of Cdk1 might not be available in the absence of Cdk2 due to higher levels of p21 available to inhibit all of Cdk1. In support to this hypothesis, it was identified that Cdk1 is essential for DNA double strand break repair in yeast [25]. Whether Cdk1 plays a similar role in mammalian cells still needs to be explored.

In contrast to Cdk2's role in the activation of DNA repair pathways it was also shown that Cdk2 phosphorylates FOXO1 in response to chemically induced DNA damage [26]. FOXO transcription factors induce apoptosis by activating a number of genes including Fas, Bim etc. [27]. In this study, a connection between Cdk2 and DNA repair was not detected but they found that FOXO1 phosphorylation and activation by Cdk2 is one of the mechanisms that triggers apoptosis when there is extensive DNA damage in response to chemical treatment that cannot be repaired. This further complicates the role of Cdk2 in DNA damage response. How does Cdk2 distinguish between moderate and extensive DNA damage? Which upstream signaling directs Cdk2 to decide whether to activate Chk1 to repair it or FOXO1 to trigger apoptosis? Although preliminary information suggests a role of Cdk2 in DNA repair in higher eukaryotic cells, it seems that a thorough analysis will be necessary in order to define the specific roles of Cdk2 in DNA repair.

## Concluding remarks

From a limited number of studies conducted so far, it emerges that Cdk2 is necessary for proper DNA repair. Nevertheless understanding the specific functions of Cdk2 in DNA repair appears to be challenging due to the complexity of the DNA damage signaling pathway. The most important question to answer is how are Cdk2/cyclin E and/or Cdk2/cyclin A complexes involved in DNA repair when they are present in an inhibited state in response to DNA damage. Although Cdk1 can fully compensate for S phase functions of Cdk2, it fails to compensate for Cdk2's DNA repair functions in mammalian cells. Moreover in addition to Cdk2's own DNA repair functions, Cdk1's specific role in mammalian DNA damage repair will also need to be resolved.

## References

[B1] Morgan DO (1997). Cyclin-dependent kinases: engines, clocks, and microprocessors. Annu Rev Cell Dev Biol.

[B2] Sherr CJ, Roberts JM (1999). Cdk inhibitors: positive and negative regulators of G1-phase progression. Genes Dev.

[B3] Sherr CJ, Roberts JM (2004). Living with or without cyclins and cyclin-dependent kinases. Genes Dev.

[B4] Weinberg RA (1995). The Retinoblstoma protein and cell cycle control. Cell.

[B5] Dyson N (1998). The regulation of E2F by pRB-family proteins. Genes & Dev.

[B6] Riabowol K, Draetta G, Brizuela L, Vandre D, Beach D (1989). The cdc2 kinase is a nuclear protein that is essential for mitosis in mammalian cells. Cell.

[B7] Heuvel S van den, Harlow E (1993). Distinct roles for cyclin-dependent kinases in cell cycle control. Science.

[B8] Aleem E, Kiyokawa H, Kaldis P (2005). Cdc2-cyclin E complexes regulate the G1/S phase transition. Nat Cell Biol.

[B9] Berthet C, Aleem E, Coppola V, Tessarollo L, Kaldis P (2003). Cdk2 knockout mice are viable. Curr Biol.

[B10] Ortega S, Prieto I, Odajima J, Martin A, Dubus P, Sotillo R, Barbero JL, Malumbres M, Barbacid M (2003). Cyclin-dependent kinase 2 is essential for meiosis but not for mitotic cell division in mice. Nat Genet.

[B11] Santamaria D, Barriere C, Cerqueira A, Hunt S, Tardy C, Newton K, Caceres JF, Dubus P, Malumbres M, Barbacid M (2007). Cdk1 is sufficient to drive the mammalian cell cycle. Nature.

[B12] O'Connor PM (1997). Mammalian G1 and G2 phase checkpoints. Cancer Surv.

[B13] Vaziri H, Benchimol S (1996). From telomere loss to p53 induction and activation of a DNA-damage pathway at senescence: the telomere loss/DNA damage model of cell aging. Exp Gerontol.

[B14] Aprelikova O, Xiong Y, Liu ET (1995). Both p16 and p21 families of cyclin-dependent kinase (CDK) inhibitors block the phosphorylation of cyclin-dependent kinases by the CDK-activating kinase. J Biol Chem.

[B15] Toyoshima H, Hunter T (1994). p27, a novel inhibitor of G1 cyclin-cdk protein kinase activity, is related to p21. Cell.

[B16] Weinert TA, Hartwell LH (1988). The RAD9 gene controls the cell cycle response to DNA damage in Saccharomyces cerevisiae. Science.

[B17] Giono LE, Manfredi JJ (2006). The p53 tumor suppressor participates in multiple cell cycle checkpoints. J Cell Physiol.

[B18] O'Connell MJ, Cimprich KA (2005). G2 damage checkpoints: what is the turn-on?. J Cell Sci.

[B19] Satyanarayana A, Hilton MB, Kaldis P (2008). p21 Inhibits Cdk1 in the Absence of Cdk2 to Maintain the G1/S Phase DNA Damage Checkpoint. Mol Biol Cell.

[B20] Esashi F, Christ N, Gannon J, Liu Y, Hunt T, Jasin M, West SC (2005). CDK-dependent phosphorylation of BRCA2 as a regulatory mechanism for recombinational repair. Nature.

[B21] Thorslund T, West SC (2007). BRCA2: a universal recombinase regulator. Oncogene.

[B22] Muller-Tidow C, Ji P, Diederichs S, Potratz J, Baumer N, Kohler G, Cauvet T, Choudary C, Meer T Van Der, Chan WY (2004). The cyclin A1-Cdk2 complex regulates DNA double-strand break repair. Mol Cell Biol.

[B23] Deans AJ, Khanna KK, McNees CJ, Mercurio C, Heierhorst J, McArthur GA (2006). Cyclin-dependent kinase 2 functions in normal DNA repair and is a therapeutic target in BRCA1-deficient cancers. Cancer Res.

[B24] Sanchez Y, Bachant J, Wang H, Hu F, Liu D, Tetzlaff M, Elledge SJ (1999). Control of the DNA damage checkpoint by Chk1 and Rad53 protein kinases through distinct mechanisms. Science.

[B25] Ira G, Pellicioli A, Balijja A, Wang X, Fiorani S, Carotenuto W, Liberi G, Bressan D, Wan L, Hollingsworth NM (2004). DNA end resection, homologous recombination and DNA damage checkpoint activation require CDK1. Nature.

[B26] Huang H, Regan KM, Lou Z, Chen J, Tindall DJ (2006). CDK2-Dependent Phosphorylation of FOXO1 as an Apoptotic Response to DNA Damage. Science.

[B27] Fu Z, Tindall DJ (2008). FOXOs, cancer and regulation of apoptosis. Oncogene.

